# Rigorous packing of unit squares into a circle

**DOI:** 10.1007/s10898-018-0711-5

**Published:** 2018-10-03

**Authors:** Tiago Montanher, Arnold Neumaier, Mihály Csaba Markót, Ferenc Domes, Hermann Schichl

**Affiliations:** 1 Wolfgang Pauli Institute, Oskar-Morgenstern-Platz 1, 1090 Vienna, Austria; 2 Faculty of Mathematics, University of Vienna, Oskar-Morgenstern-Platz 1, 1090 Vienna, Austria

**Keywords:** Square packing into a circle, Interval branch-and-bound, Tiling constraints, Computer-assisted proof, 52C15, 90C26, 65K05, 65G30

## Abstract

This paper considers the task of finding the smallest circle into which one can pack a fixed number of non-overlapping unit squares that are free to rotate. Due to the rotation angles, the packing of unit squares into a container is considerably harder to solve than their circle packing counterparts. Therefore, optimal arrangements were so far proved to be optimal only for one or two unit squares. By a computer-assisted method based on interval arithmetic techniques, we solve the case of three squares and find rigorous enclosures for every optimal arrangement of this problem. We model the relation between the squares and the circle as a constraint satisfaction problem (CSP) and found every box that may contain a solution inside a given upper bound of the radius. Due to symmetries in the search domain, general purpose interval methods are far too slow to solve the CSP directly. To overcome this difficulty, we split the problem into a set of subproblems by systematically adding constraints to the center of each square. Our proof requires the solution of 6, 43 and 12 subproblems with 1, 2 and 3 unit squares respectively. In principle, the method proposed in this paper generalizes to any number of squares.

**Electronic supplementary material:**

The online version of this article (10.1007/s10898-018-0711-5) contains supplementary material, which is available to authorized users.

## Introduction

Let $S_{1},\ldots , S_{n}$ be *n* open unit squares and denote by $C_{r}$ the closed circle of radius *r* centered at the origin. This paper deals with the problem of finding the smallest value of *r* such that one can pack $S_{1},\ldots , S_{n}$ into $C_{r}$ without overlapping. Formally, we can write the problem as

1 $$\begin{aligned} \begin{aligned}&\min ~~ r \\&{\mathrm{s.t.~}}~~~S_{i} \subseteq C_{r} \quad 1 \le i \le n\\&\qquad ~~~ S_{i} \cap S_{j} = \emptyset \quad 1 \le i,j \le n, ~~ i \ne j. \end{aligned} \end{aligned}$$

Packing identical objects into a container is an attractive part of geometrical optimization. The subject drew the attention of a considerable number of researchers, who contributed to problems similar to the one discussed in this paper.

The circle packing is the simplest packing problem in 2 dimensions in the sense that it does not involve the angles of the objects. Markót studied the packing of circles into a square from the interval analysis point of view in a series of papers [15, 16, 24]. In particular, he proved rigorous bounds for $n = 28, 29$ and 30 circles. For a survey of the circle packing under the global optimization point of view, see [5]. The website *Packomania* [22] maintains an updated list of the best-known values for the packing of equal circles into several containers.

Kallrath and Rebennack [12] studied the packing of ellipses into rectangles using state-of-the-art complete global optimization solvers. He succeeded to find the global optimum for the case $n = 3$ without rigor. For the packing of ellipsoids, see [2, 3].

Erdös and Graham [8] inaugurated the packing of unit squares into a square. They show that the wasted area in a container with side length *l* is $O(l^{\frac{7}{11}})$. The proof relies on geometrical arguments and not on rigorous computations. Recent contributions in the packing of unit squares into a square include new bounds for the wasted area [6], the optimality proof for the cases $n = 5,\ldots ,10, 13$ and 46 [1, 10, 23] and the optimality proof for $n-2$ and $n-1$ whenever *n* is a square [19]. Again, none of these contributions rely on computer-assisted proofs. For a dynamic survey on the packing of unit squares in a square, see [10].

The packing of unit squares into general containers received considerably less attention than the circle or the unit square packing into a square. For example, Friedman [9] maintains a list of proved and best-known values for the packing of unit squares into circles, triangles, L-shapes, and pentagons. In each case, only trivial arrangements are proved optimal. For the subject of interest in this paper, the packing of unit squares into a circle, the first open case is $n = 3$. For a list of figures of squares packed into a circle, see https://www2.stetson.edu/~efriedma/squincir/.

### Contribution and outline

This paper introduces a computer-assisted method for finding rigorous enclosures for *r* in Problem (1) and the corresponding optimal arrangements. The method is of theoretical interest since it proves optimality instead of only presenting a feasible arrangement. Therefore, it is suitable for small values of *n* only.

Our approach relies on the interval branch-and-bound framework. We implement the algorithm in *C++* using the forward-backward constraint propagation [21] to reduce the search domain. Section 2 introduces the solver. The code is available at http://www.mat.univie.ac.at/~montanhe/publications/n3.zip.

Section 3 formulates Problem (1) as a constraint satisfaction problem (CSP). This paper uses the concept of sentinels [4, 18] to model non-overlapping conditions and the convexity of the circle to write containment constraints. Given an upper bound $\overline{r}_{n}$ for $r_{n}$, the CSP asks for every feasible arrangement satisfying $r \le \overline{r}_{n}$. Our software produces a list of small interval vectors with the property that every optimal arrangement of (1) belongs to at least one element in the list.

General purpose interval solvers are usually not capable of solving packing problems due to symmetries in the search domain. To overcome this difficulty, Sect. 4 shows how to split the original CSP into a set of subproblems by systematically adding constraints to the center of each square. We call them tiling constraints as the idea resembles the one proposed in [15, 16, 24]. The tiling divides the search domain into a set of isosceles triangles that must contain the center of at most one unit square. Then, one can replace the original CSP by a set of $$\left( {\begin{array}{c}K\\ n\end{array}}\right) $$ subproblems, where *K* is the number of triangles in the tiling.

Our procedure iterates on the number of squares to avoid the exponential growth of subproblems. At the *i*-th iteration, we look at every possible combination of *i* triangles which can accommodate *i* unit squares into a circle with the radius at most $\overline{r}_{n}$. The rationale behind this strategy is twofold: (i) It allows us to discard a large number of hard subproblems by proving the infeasibility of more straightforward cases and (ii) It propagates the reduction on the search domain through the iterations. We also show that some combinations of triangles are symmetric by construction. Then one can discard them without any processing. This observation in addition to our iterative method reduces the number of hard cases considerably.

Section 5 illustrates the capabilities of our method. We find a mathematically rigorous enclosure for $r_{3}$ and the corresponding optimal arrangement. If one set $\overline{r} = \frac{5\sqrt{17}}{16}$ as pointed by Friedman [9], the tiling produces 36 triangles. Our approach requires the solution of 6 subproblems with one square, 43 with two and only 12 subproblems with 3 squares to conclude the proof. It is $<\,1\%$ of all possible $$\left( {\begin{array}{c}36\\ 3\end{array}}\right) = 7140$$ combinations. The method could also be used to find optimal configurations for higher values of *n* (e.g., $n = 4, 5, 6$).

### Interval notation

This paper is an application of the interval branch-and-bound framework [11, 13]. We assume that the reader is familiar with concepts from interval analysis [20]. Let $\underline{a}, \overline{a} \in \mathbb {R}$ with $\underline{a} \le \overline{a}$. Then $\mathbf{a}=[\underline{a}, \overline{a}]$ denotes the interval with $\inf (\mathbf{a}) := \min (\mathbf{a}) := \underline{a}$ and $\sup (\mathbf{a}) := \max (\mathbf{a}) := \overline{a}$. We denote the width of the interval $\mathbf{a}$ by ${\mathrm{wid}}(\mathbf{a}) := \overline{a} - \underline{a}$.

The set of nonempty compact real intervals is given by

$$\begin{aligned} \mathbb {I}\mathbb {R}:=\{[\underline{a}, \overline{a}] \mid \underline{a} \le \overline{a},~ \underline{a}, \overline{a} \in \mathbb {R}\}. \end{aligned}$$

Let $S \subseteq \mathbb {R}$ be any set. Then the interval hull  of *S* is the smallest interval containing *S*.

An interval vector (also called box) $\mathbf{x}:= [\underline{x},\overline{x}]$ is the Cartesian product of the closed real intervals $\mathbf{x}_i:=[\underline{x}_i, \overline{x}_i] \in \mathbb {IR}$. We denote the set of all interval vectors of dimension *n* by $\mathbb {IR}^{n}$. We apply the width operator component wise on vectors. Therefore $\max ({\mathrm{wid}}(\mathbf{x})) := \max ({\mathrm{wid}}(\mathbf{x}_{1}),\ldots ,{\mathrm{wid}}(\mathbf{x}_{n}))$. Interval operations and functions are defined as in [13, 20]. The absolute value of the interval $\mathbf{a}$ is given by

$$\begin{aligned} |\mathbf{a}| :=\left\{ \begin{array}{ll} \mathbf{a}&{}\quad \text{ if } \inf (\mathbf{a}) \ge 0 ,\\ {[}0, \max (-\inf (\mathbf{a}), \sup (\mathbf{a})){]} &{}\quad \text{ if } 0 \in \mathbf{a},\\ -\mathbf{a}&{}\quad \text{ if } \sup (\mathbf{a}) \le 0. \end{array}\right. \end{aligned}$$

Let $\mathbf{a}$ and $\mathbf{b}$ be two intervals. The maximum of $\mathbf{a}$ and $\mathbf{b}$ is defined by

$$\begin{aligned} \max (\mathbf{a}, \mathbf{b}) := [\max (\inf (\mathbf{a}), \inf (\mathbf{b})), \max (\sup (\mathbf{a}), \sup (\mathbf{b}))]. \end{aligned}$$

Let $F:\mathbb {R}^{n} \rightarrow \mathbb {R}^{m}$ be a function defined on $\mathbf{x}\in \mathbb {IR}^{n}$ and let $\mathbf{f}\in \mathbb {IR}^{m}$. We denote the natural interval extension of the function *F* by $\mathbf{F}$. A constraint satisfaction problem (CSP) is the task of finding every point satisfying

$$\begin{aligned} F(x) \in \mathbf{f},~~ x \in \mathbf{x}. \end{aligned}$$

We call $\mathbf{x}$ the search domain and the problem is said to be infeasible if there is no $x \in \mathbf{x}$ satisfying $F(x) \in \mathbf{f}$. We also denote constraint satisfaction problems by the triplet $(F, \mathbf{f}, \mathbf{x})$.

## The algorithm

This section describes the algorithm designed for solving the subproblems of form (1) using interval arithmetic [11, 13, 20] The solver consists of two components, the memory, and the reducer. The former manages the branch-and-bound tree while the latter is responsible for processing the current box. There is also a post-processing step called cluster builder to group close boxes in the solution list.

The memory keeps the list of unprocessed boxes. It is also responsible for the box selector, and to split the boxes coming from the reducer that cannot be discarded or saved as a solution. In this paper, the selector is a depth-first search procedure while the splitter creates two boxes by dividing the input in the midpoint of the coordinate with maximum width.

The reducer contains a list of rigorous methods to reduce or discard boxes. This paper uses the forward-backward constraint propagation [21] and a feasibility verification method [7]. We consider a CSP of form $(F, \mathbf{x}, \mathbf{f})$ in the next paragraphs to overview each method.

The forward-backward constraint propagation decomposes $\mathbf{F}$ into a set of simple functions (like the exponential function or the sum of several elements) and displays the pieces in a graph. The forward step is a procedure to evaluate $\mathbf{F}(\mathbf{x})$ systematically. In this case, the data flow from the decision variable nodes of the graph to the constraint nodes $\mathbf{F}_{1},\ldots , \mathbf{F}_{m}$. At the end of this step, each constraint node contains an enclosure of $\mathbf{F}_{i}(\mathbf{x}) \cap \mathbf{f}_{i}$. The backward step acts reversely. It starts from the constraint nodes $\mathbf{F}(\mathbf{x})\cap \mathbf{f}$ and walks the graph applying inverse functions until reaching $\mathbf{x}_{1},\ldots , \mathbf{x}_{n}$. At the end of the backward step, we have a new box $\mathbf{x}' \subseteq \mathbf{x}$ with the reduced search domain.

This paper employs the following feasibility verification method. Let $\mathbf{x}$ be a box and define the midpoint of $\mathbf{x}$ as $x^{*}$. Then, we build a small box $\mathbf{x}^{*}$ around the $x^{*}$ and check its feasibility. The box $\mathbf{x}^{*}$ is a feasible if $\mathbf{F}(\mathbf{x}^{*}) \subseteq \mathbf{f}$. We also save a box $\mathbf{x}$ as solution if it satisfies $\max ({\mathrm{wid}}(\mathbf{x})) < \epsilon _{x}$ for a given $\epsilon _{x} > 0$.

The order into which we call the rigorous methods to process $\mathbf{x}$ may influence the efficiency of the branch-and-bound procedure. In this paper, the methods follow the finite state machine described in Table 1.

**Table 1:** The finite state machine for the inner loop of the Algorithm 1

Current state	Next state	Condition
Forward CP [21]	Exit	Box $\mathbf{x}$ is infeasible
	Backward CP	Otherwise
Backward CP [21]	Forward CP	$G_{Rel}(\mathbf{x}, \mathbf{x}') > \epsilon _{T}$
	Feasibility verification	Otherwise
Feasibility verification [7]	Exit	True

The parameter $\epsilon _{T} > 0$ is the threshold tolerance which controls the relative gain of the box $\mathbf{x}' \subseteq \mathbf{x}$ with the help of the following function

$$\begin{aligned} G_{Rel}(\mathbf{x}, \mathbf{x}') := \max _{\begin{array}{c} {i=1,\ldots ,n;}\\ {{\mathrm{wid}}(\mathbf{x}_{i}) > 0} \end{array}}\left( \frac{{\mathrm{wid}}(x'_{i})}{{\mathrm{wid}}(\mathbf{x}_{i})}\right) . \end{aligned}$$

It is clear that the input of $G_{Rel}$ at each iteration is the box $\mathbf{x}$ and the outcome of the rigorous method, $\mathbf{x}'$.

After processing every box in the memory, we run a post-processing step to build clusters of solutions. This method supports the analysis of the solution list since it reduces the number of boxes on it. Given two intervals $\mathbf{a}$ and $\mathbf{b}$, we define the gap between $\mathbf{a}$ and $\mathbf{b}$ by

$$\begin{aligned} \text {gap}(\mathbf{a}, \mathbf{b}) :=\left\{ \begin{array}{ll} \inf (\mathbf{b}) - \sup (\mathbf{a}) &{}\quad \text{ if } \inf (\mathbf{b})> \sup (\mathbf{a}) ,\\ \inf (\mathbf{a}) - \sup (\mathbf{b}) &{}\quad \text{ if } \inf (\mathbf{a}) > \sup (\mathbf{b}) ,\\ 0 &{}\quad \text{ if } \mathbf{a}\cap \mathbf{b}\ne \emptyset . \end{array}\right. \end{aligned}$$

We save two boxes $\mathbf{x}, \mathbf{y}\in \mathbb {IR}^{n}$ in the same group if

$$\begin{aligned} \max _{i=1,\ldots , n} \text {gap}(\mathbf{x}_{i}, \mathbf{y}_{i}) < \epsilon _{C} \end{aligned}$$

where $\epsilon _{C}$ is the cluster builder tolerance. After assigning a group to every box in the solution set, we return the interval hull of each group and conclude the procedure.

Algorithm 1 summarizes the interval branch-and-bound method. We implement the algorithm in *C++* using two interval arithmetic libraries, the *Filib* [14] and the *Moore* [17]. The user can choose any of these implementations in the verification of the proof. We report only results from the test with *Filib* in this paper. The supplementary material also reports the results utilizing *Moore*. They are consistent with each other.
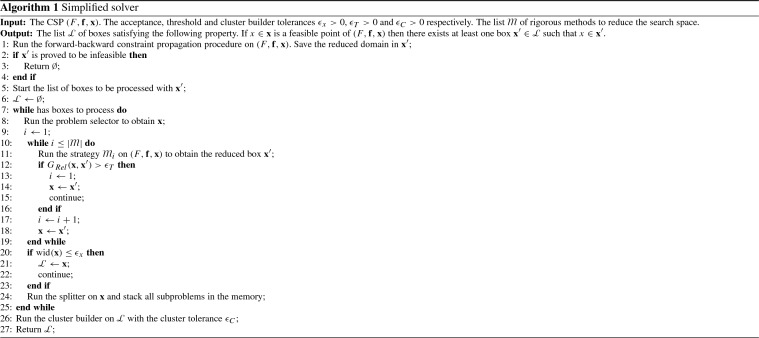


## The standard model

This section introduces the mathematical model for the containment and the non-overlapping conditions of (1). We call the resulting model the standard constraint satisfaction problem since it is the same for every subproblem. We assume that the squares have side length *s*. The inequalities for the containment condition follow from the convexity of the circle. On the other hand, non-overlapping constraints rely on the concept of sentinels [4, 18].

### Containment

Let $C_{r}$ be the closed circle of radius *r* and centered at the origin. The convexity of the circle implies that $c \in C_{r}$ for any point *c* in the segment of line $\overline{ab}$ if $a, b \in C_{r}$. Then, a given square belongs to $C_{r}$ if and only if its vertices belong to $C_{r}$.

Let $S_{0,0}$ be the open square centered at the origin, with no rotation angle and side length *s*. Then

$$\begin{aligned} S_{0,0} := \big \{ x \in \mathbb {R}^{2} \mid \max (|x_{1}|,|x_{2}| ) - \frac{s}{2} < 0 \big \}. \end{aligned}$$

We denote the closure of a set *S* by $\overline{S}$. The set of vertices of $\overline{S}_{0, 0}$ is given by

$$\begin{aligned} V_{0, 0} := \{V^{NW}, V^{SW}, V^{NE}, V^{SE}\} \end{aligned}$$

where

$$\begin{aligned} V^{NW} := \left( \begin{array}{c} -\frac{s}{2} \\ \frac{s}{2}\\ \end{array} \right) , V^{SW} := \left( \begin{array}{c} -\frac{s}{2} \\ -\frac{s}{2}\\ \end{array} \right) , V^{NE} := \left( \begin{array}{c} \frac{s}{2} \\ \frac{s}{2}\\ \end{array} \right) , V^{SE} := \left( \begin{array}{c} \frac{s}{2} \\ -\frac{s}{2}\\ \end{array} \right) . \end{aligned}$$

For any $c \in \mathbb {R}^{2}$ and $\theta \in \mathbb {R}$, we define the displacement operator as

2 $$\begin{aligned} h(c, \theta , x) := c + A_{\theta }x \end{aligned}$$

where $A_{\theta }$ is the rotation matrix

$$\begin{aligned} A_{\theta } := \left( \begin{array}{cc} \cos (\theta )&{} -\sin (\theta ) \\ \sin (\theta ) &{} \cos (\theta )\\ \end{array} \right) . \end{aligned}$$

The open square centered at $c \in \mathbb {R}^{2}$, with rotation angle $\theta \in [0, \frac{\pi }{2})$ and side length *s* is the set given by

3 $$\begin{aligned} S_{c, \theta } := \big \{ z \in \mathbb {R}^{2} \mid z = h(c, \theta , x), ~ x \in S_{0, 0} \big \}. \end{aligned}$$

The set of vertices of $\overline{S}_{c, \theta }$, denoted by $V_{c, \theta }$, is the union of the following points

$$\begin{aligned} V_{c, \theta }^{P} := c + A_{\theta }V^{P}, ~~ P \in \{NW, SW, NE, SE\}. \end{aligned}$$

Finally, we denote the circle of radius *r* and centered at the origin by $C_{r}$. Then

$$\begin{aligned} C_{r} := \{x \in \mathbb {R}^{2} \mid x_{1}^{2} + x_{2}^{2} \le r^{2} \}. \end{aligned}$$

#### Proposition 1

Let $g_{r}(x) := x_{1}^2 + x_{2}^2 - r^{2}$ and consider the following inequalities

4 $$\begin{aligned} g_{r}(V_{c, \theta }^{P}) \le 0, ~~ P \in \{NW, SW, NE, SE\} \end{aligned}$$

Then

$$\begin{aligned} \overline{S}_{c, \theta } \subseteq \mathbf{C}_{r} ~~~\Leftrightarrow ~~~(4) \text { hold.} \end{aligned}$$

#### Proof

If $\overline{S}_{c, \theta } \subseteq C_{r}$ then $V_{c, \theta } \subseteq C_{r}$ and (4) hold. Conversely, since $\overline{S}_{c, \theta }$ is a bounded polytope, it is given by the convex hull of the elements of $V_{c, \theta }$. The result follows from the convexity of the circle. $\square$

### Non-overlapping

**Fig. 1:**
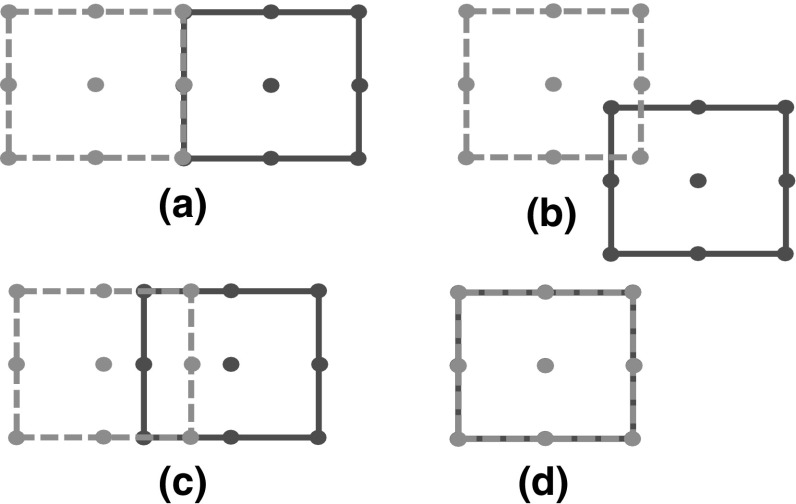
**a** Non-overlapping squares. **b** Vertex sentinel violation. **c** Mid-point sentinel violation. **d** Center sentinel violation

This subsection shows that two squares $S_{c_{1},\theta _{1}}$ and $S_{c_{2},\theta _{2}}$ are non-overlapping if and only a set of nine points defined on $S_{c_{1},\theta _{1}}$ do not belong to $S_{c_{2},\theta _{2}}$ and vice-versa. We call such sets sentinels of a square. Figure 1 illustrates the need of the sentinels in the non-overlapping formulation.

The set of sentinels of $S_{0, 0}$ is given by

$$\begin{aligned} T_{0,0} := V_{0,0} \cup \{ V^{N}, V^{S}, V^{E}, V^{W}, V^{O}\} \end{aligned}$$

where

$$\begin{aligned} V^{N} := \left( \begin{array}{c} 0 \\ \frac{s}{2} \\ \end{array} \right) , V^{S} := \left( \begin{array}{c} 0 \\ -\frac{s}{2} \\ \end{array} \right) , V^{E} := \left( \begin{array}{c} \frac{s}{2} \\ 0 \\ \end{array} \right) , V^{W} := \left( \begin{array}{c} -\frac{s}{2} \\ 0 \\ \end{array} \right) , V^{O} := \left( \begin{array}{c} 0 \\ 0 \\ \end{array} \right) . \end{aligned}$$

We denote the set of sentinels of $S_{c, \theta }$ by $T_{c, \theta }$. This set is given by the union of the following points

$$\begin{aligned} T_{c, \theta }^{P} := c + A_{\theta }V^{P}, ~~ P \in \{NW, SW, NE, SE, N, S, E, W, O\}. \end{aligned}$$

The next theorem states that the non-overlapping condition between two squares reduces to the containment verification of their sets of sentinels. It is a particular case of the sentinels theorem proved in [18].

#### Theorem 1

Let $S_{c_{i}, \theta _{i}}$ and $S_{c_{j}, \theta _{j}}$ be two squares defined by (3) and let $T_{c_{i}, \theta _{i}}$ and $T_{c_{j}, \theta _{j}}$ be their corresponding sets of sentinels. Then

$$\begin{aligned} S_{c_{i}, \theta _{i}} \cap S_{c_{j}, \theta _{j}} = \emptyset ~~~\Leftrightarrow ~~~S_{c_{i}, \theta _{i}} \cap T_{c_{j}, \theta _{j}} = \emptyset \text { and } S_{c_{j}, \theta _{j}} \cap T_{c_{i}, \theta _{i}} = \emptyset . \end{aligned}$$

In order to check conditions of form $S_{c_{i}, \theta _{i}} \cap T_{c_{j}, \theta _{j}} = \emptyset$ numerically, we need the definition of the inverse of the displacement operator (2)

$$\begin{aligned} h^{-1}(c, \theta , z) := A_{\theta }^{T}(z - c). \end{aligned}$$

#### Lemma 1

Let $z \in \mathbb {R}^{2}$ and $S_{c, \theta }$ be a square defined by (3). Then

$$\begin{aligned} z \in S_{c, \theta } ~~~\Leftrightarrow ~~~\max (|h_{1}^{-1}(c, \theta , z)|, |h_{2}^{-1}(c, \theta , z)|) - \frac{s}{2} < 0. \end{aligned}$$

where $h_{1}^{-1}$ and $h_{2}^{-1}$ are the coordinates of the inverse operator.

#### Proof

If $z \in S_{c, \theta }$ then there exists $x \in S_{0,0}$ such that $x = h^{-1}(c, \theta , z)$ and the implication follows immediately. Conversely, let $x := h^{-1}(c, \theta , z)$. The left hand side of the equivalence implies that $x \in S_{0,0}$. If we let $z' := c + A_{\theta }x$ then $z' = c + A_{\theta }A_{\theta }^{T}(z - c) = z$. Therefore $z \in S_{c, \theta }$ and the result follows. $\square$

Applying the inverse of the displacement operator of the square $S_{c_{i}, \theta _{i}}$ to the point $T_{c_{j}, \theta _{j}}^{P} \in T_{c_{j}, \theta _{j}}$ gives

5 $$\begin{aligned} h^{-1}(c_{i}, \theta _{i}, T_{c_{j}, \theta _{j}}^{P}) = A^{T}_{\theta _{i}}(c_{j} + A_{\theta _{j}}V^{P} - c_{i}), ~~ V^{P} \in T_{0,0}. \end{aligned}$$

Let $c_{j,1}$ and $c_{j,2}$ be the coordinates of the vector $c_{j}$. Then the coordinates of (5) are given by

$$\begin{aligned} u_{1}(c_{i}, c_{j}, \theta _{i}, \theta _{j}, V):= & {} \cos (\theta _{i})(c_{j,1} - c_{i,1}) - \sin (\theta _{i})(c_{j,2} - c_{i,2}) \\&+ \cos (\theta _{i} - \theta _{j})V_{1} + \sin (\theta _{i} - \theta _{j})V_{2} \end{aligned}$$

and

$$\begin{aligned} u_{2}(c_{i}, c_{j}, \theta _{i}, \theta _{j}, V):= & {} \sin (\theta _{i})(c_{j,1} - c_{i,1}) + \cos (\theta _{i})(c_{j,2} - c_{i,2}) +\\&- \cos (\theta _{i} - \theta _{j})V_{1} + \cos (\theta _{i} - \theta _{j})V_{2}. \end{aligned}$$

The following proposition shows that the verification of $S_{c_{i}, \theta _{i}} \cap T_{c_{j}, \theta _{j}} = \emptyset$ reduces to the evaluation of nine non-smooth functions.

#### Proposition 2

Let $S_{c_{i}, \theta _{i}}$ and $T_{c_{j}, \theta _{j}}$ be as in Theorem 1 and define the function

$$\begin{aligned} u(c_{i}, c_{j}, \theta _{i}, \theta _{j}, V^{P}) := \max (|u_{1}(c_{i}, c_{j}, \theta _{i}, \theta _{j}, V^{P})|, |u_{2}(c_{i}, c_{j}, \theta _{i}, \theta _{j}, V^{P})|). \end{aligned}$$

Then

$$\begin{aligned} S_{c_{i}, \theta _{i}} \cap T_{c_{j}, \theta _{j}} = \emptyset ~~~\Leftrightarrow ~~~u(c_{i}, c_{j}, \theta _{i}, \theta _{j}, V^{P}) - \frac{s}{2} \ge 0 ~\text { for all } V^{P} \in T_{0,0}. \end{aligned}$$

#### Proof

Follows from the application of the Lemma 1 to the elements of $T_{c_{j}, \theta _{j}}$. $\square$

### The standard model

We conclude this section with the formal statement of the standard constraint satisfaction problem. Here and throughout we assume, without loss of generality, that the angle of the first square is always 0. This condition follows from the proper rotation of the remaining squares into the circle.

#### Definition 1

[SCSP] Let $\overline{r} > 0$ be an upper bound for the radius of the smallest circle into which one can pack *n* non-overlapping unit squares and *s* be a scaling factor. We denote the following problem by standard constraint satisfaction problem (SCSP)

6 $$\begin{aligned} \text {find}&(r, c_{1},\theta _{1},\ldots ,c_{n},\theta _{n}) \\ {\mathrm{s.t.~}}&u(c_{i}, c_{j}, \theta _{i}, \theta _{j}, V^{P}) - \frac{s}{2} \ge 0 \nonumber \\&g_{r}( V_{c_{i}, \theta _{i}}^{P} ) \le 0 \nonumber \\&c_{i,1}, c_{i,2} \in [- r, r] \nonumber \\&\theta _{i} \in [0, \frac{\pi }{2}] \nonumber \\&\theta _{1} = 0 \nonumber \\&r \le \overline{r} \nonumber \end{aligned}$$

where $i,j = 1,\ldots , n$ with $i \ne j$, $V^{P} \in T_{0,0}$ and $V_{c_{i}, \theta _{i}}^{P} \in V_{c_{i},\theta _{i}}$. Functions $g_{r}$ and *u* are given by Propositions 1 and 2 respectively.

## Tiling

General purpose interval branch-and-bound procedures cannot solve the SCSP in a reasonable amount of time even for small values of *n* due to symmetries in the search space. This section introduces a tiling method to split (6) into a set of subproblems suitable for the Algorithm 1.

We employ the *Matlab*-like notation $g := a:s:b$ to denote the array with $k := \lfloor \frac{b - a}{s} \rfloor + 1$ elements where $g_{i} := a + i s$ for $i = 0,\ldots , k - 1$. In addition, we denote the array with the midpoints of *g* by $g_{c}$. Then,

$$\begin{aligned} g_{c,i} := \frac{g_{i} + g_{i+1}}{2}, ~~ i = 0,\ldots , k - 2. \end{aligned}$$

Let $\overline{r} > 0$ be an upper bound for the SCSP. Then, the step length

7 $$\begin{aligned} l := \frac{2 \overline{r}}{\lfloor 2 \overline{r} + 1 \rfloor } \end{aligned}$$

splits $[-\overline{r}, \overline{r}]$ into $\lfloor 2 \overline{r} + 1 \rfloor$ equally divided intervals. Let $V := \{v \in \mathbb {R}\mid v = -\overline{r} + il, i \in \mathbb {Z}\} \cap [-\overline{r}, \overline{r}]$ be the end points of each interval, satisfying $v_{i} := -\overline{r} + i l$ for $i = 0,\ldots , p : = \lfloor 2 \overline{r} + 1 \rfloor$. Moreover, we write the midpoints of *V* as $V_{c}$ where $v_{c, i} := v_{i} + \frac{l}{2}$ for $i = 0,\ldots , p-1$. Let  and . We denote the elements of  by $$ v_{i,j} := \left( \begin{array}{c} v_{i}\\ v_{j}\\ \end{array} \right) $$ for $v_{i}, v_{j} \in v$ and $0 \le i, j \le p$. In the same way, we write the elements of  as $$ c_{i,j} := v_{i,j} + \left( \begin{array}{c} \frac{l}{2}\\ \frac{l}{2}\\ \end{array} \right) $$ for  and $0 \le i, j \le p - 1$. Algorithm 2 produces the sets  and .
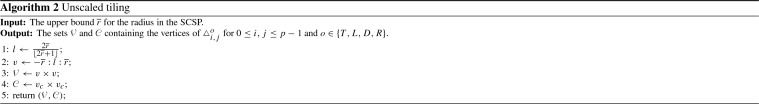


Let $\triangle ABC$ be the triangle with vertices $A, B, C \in \mathbb {R}^{2}$. Then, we define the following triangles for $0 \le i, j \le p -1$

$$\begin{aligned} \triangle _{i,j}^{T}:= & {} \triangle v_{i,j+1} v_{i+1,j+1} c_{i,j}, \\ \triangle _{i,j}^{L}:= & {} \triangle v_{i,j+1} v_{i,j} c_{i,j}, \\ \triangle _{i,j}^{D}:= & {} \triangle v_{i,j} v_{i+1,j} c_{i,j}, \\ \triangle _{i,j}^{R}:= & {} \triangle v_{i+1,j} v_{i+1,j+1} c_{i,j}. \end{aligned}$$

**Fig. 2:**
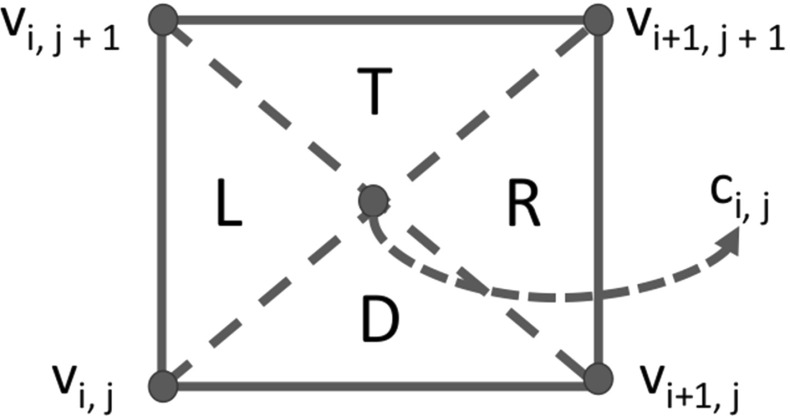
The geometrical meaning of $\triangle _{i,j}^{o}$ for $0 \le i, j \le p -1$ and $o \in \{T, L, D, R\}$

Here, *T*, *L*, *D* and *R* stand for top, left, down and right respectively. Figure 2 shows that the definition aims to split the square with vertices $v_{i,j}$, $v_{i+1,j}$, $v_{i+1, j+1}$ and $v_{i, j+1}$ into four triangles. One can easily verify that the triangles can be written as

8 $$\begin{aligned} \triangle _{i,j}^{T}:= & {} \{x \in \mathbb {R}^{2} \mid x_{2} - x_{1} \ge g_{j} - g_{i}, ~ x_{2} + x_{1} \ge g_{i} + g_{j+1}, \nonumber \\&x_{1} \in [g_{i}, g_{i+1}] , ~ x_{2} \in [g_{j} + \frac{l}{2}, g_{j+1}]\}, \end{aligned}$$

9 $$\begin{aligned} \triangle _{i,j}^{L}:= & {} \{x \in \mathbb {R}^{2} \mid x_{2} - x_{1} \ge g_{j} - g_{i}, ~ x_{2} + x_{1} \le g_{i} + g_{j+1}, \nonumber \\&x_{1} \in [g_{i}, g_{i} + \frac{l}{2}] , ~ x_{2} \in [g_{j}, g_{j+1}]\}, \end{aligned}$$

10 $$\begin{aligned} \triangle _{i,j}^{D}:= & {} \{x \in \mathbb {R}^{2} \mid x_{2} - x_{1} \le g_{j} - g_{i}, ~ x_{2} + x_{1} \le g_{i} + g_{j+1}, \nonumber \\&x_{1} \in [g_{i}, g_{i+1}] , ~ x_{2} \in [g_{j}, g_{j} + \frac{l}{2}]\}, \end{aligned}$$

11 $$\begin{aligned} \triangle _{i,j}^{R}:= & {} \{x \in \mathbb {R}^{2} s\mid x_{2} - x_{1} \le g_{j} - g_{i}, ~ x_{2} + x_{1} \ge g_{i} + g_{j+1}, \nonumber \\&x_{1} \in [g_{i} + \frac{l}{2}, g_{i+1}] , ~ x_{2} \in [g_{j}, g_{j+1}]\}. \end{aligned}$$

Lemma 2 is a collection of results needed in this section. In particular, Lemma 2-6 shows that the union of triangles $\triangle _{i, j}^{o}$ for $0 \le i, j \le p -1$ and $o \in \{T, L, D, R\}$ tiles the search domain associated to the center variables in the SCSP.

### Lemma 2

Let  and $\triangle _{i,j}^{o}$ be defined as above. Then,

1. $$l < 1$$.
2. $$x \in v ~~\Rightarrow ~-x \in v$$.
3. If  then  where
  $$\begin{aligned} v_{i,j}^{90}:= & {} \left( \begin{array}{c} -v_{j}\\ v_{i}\\ \end{array} \right) , v_{i,j}^{180} := \left( \begin{array}{c} -v_{i}\\ -v_{j}\\ \end{array} \right) , v_{i,j}^{270} := \left( \begin{array}{c} v_{j}\\ -v_{i}\\ \end{array} \right) ,\\ v_{i,j}^{x}:= & {} \left( \begin{array}{c} v_{i}\\ -v_{j}\\ \end{array} \right) , v_{i,j}^{y} := \left( \begin{array}{c} -v_{i}\\ v_{j}\\ \end{array} \right) , v_{i,j}^{Id} := \left( \begin{array}{c} v_{j}\\ v_{i}\\ \end{array} \right) , v_{i,j}^{-Id} := \left( \begin{array}{c} -v_{j}\\ -v_{i}\\ \end{array} \right) . \end{aligned}$$
4. If  then  where the vectors are defined analogously as above.
5. $\triangle _{i,j}^{o}$ is an isosceles triangle with base length *l* and legs with length $\frac{l\sqrt{2}}{2}$ for $0 \le i,j \le p -1$ and $o \in \{T, L, D, R\}$.
6. $$\begin{aligned}{}[-\overline{r}, \overline{r}]^{2} \equiv \bigcup _{\begin{array}{c} {0 \le i, j \le p} \\ {o \in \{T, L, D, R\}} \end{array}} \triangle _{i, j}^{o}. \end{aligned}$$

### Proof

1. For $a > 0$, we have $\lfloor a + 1 \rfloor = a + 1 - \delta$ where $\delta \in [0, 1)$ is the fractional part of $a + 1$. Then $1 - \delta > 0$ and $\lfloor a + 1 \rfloor > a$. The result follows by taking $a = 2 \overline{r}$.
2. If $x \in v$ then $-x = \overline{r} - il$ for some $i \in 0, \ldots , p$. Let $y = -\overline{r} + jl$ and we need to verify if there exists some $j \in 0, \ldots , p$ such that $y = -x$. The equality holds by taking $j = p - i$.
3. If  then  and the result follows from the application of Lemma 2-2 of this proposition to each case.
4. The proof is similar to the case above.
5. For $\triangle _{i, j}^{T}$, we have $\Vert v_{i,j+1} - v_{i + 1, j + 1}\Vert = l$ and
  $$\begin{aligned} \Vert v_{i,j+1} - c_{i, j}\Vert = \Vert v_{i + 1,j + 1} - c_{i, j}\Vert = \frac{l\sqrt{2}}{2}. \end{aligned}$$
  The proof is similar for $o \in \{L, D, R\}$.
6. Let $S_{i, j}$ be the closed square with vertices $v_{i,j}$, $v_{i+1, j}$, $v_{i + 1, j + 1}$, $v_{i, j + 1}$. Since $v_{0} = -\overline{r}$ and $v_{p} = \overline{r}$ it is clear that
  $$\begin{aligned}{}[-\overline{r}, \overline{r}]^{2} \equiv \bigcup _{0 \le i, j \le p - 1} S_{i, j}. \end{aligned}$$
  The result follows by noting that
  $$\begin{aligned} S_{i, j} \equiv \bigcup _{o \in \{T, L, D, R\}} \triangle _{i, j}^{o}. \end{aligned}$$
  $$\square $$

**Fig. 3:**
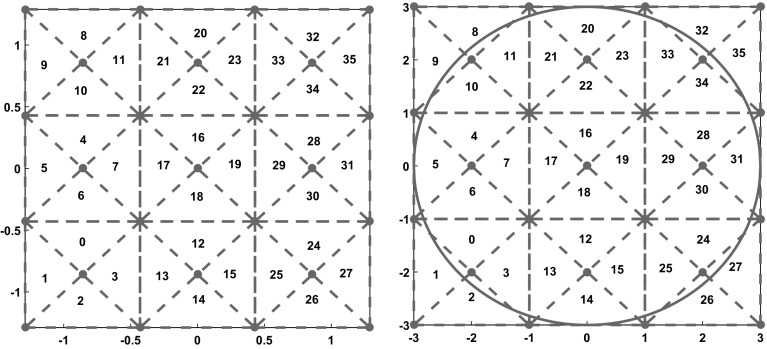
Left: Tiling for the square $[-\overline{r}_{3}, \overline{r}_{3}]^{2}$ where $\overline{r}_{3} = \frac{5\sqrt{17}}{16}$. Right: Tiling for the square $[-3, 3]^{2}$

We also assign a label to each triangle in the tiling. It helps us to easily identify a specific triangle during the proof of the case $n = 3$ in Sect. 5. Triangles of form $\triangle _{i,j}^{T}$ receive an index that is divisible by 4. In the same way, we assign labels to the left, down and right triangles with the congruence classes 1, 2 and 3 modulo 4, respectively. We denote the triangle with label *i* by $T_{i}$. Figure 3-Left shows the tiling for the best known upper bound of $r_{3}$.

We show now that each triangle of form $\triangle _{i, j}^{o}$ contains the center of at most one unit square.

### Lemma 3

The minimal distance between the centers of two non-overlapping unit squares is 1.

### Proof

Assume the contrary, let *pq* be a line segment of the centers with lower than 1. Let $C_{p}$ and $C_{q}$ the circles of radius $\frac{1}{2}$ drawn into the squares. Then $C_{p}$ and $C_{q}$ intersect. But then since the squares are supersets of $C_{p}$ and $C_{q}$, respectively, they also intersect. A contradiction. $\square$

### Proposition 3

Let $\triangle _{i, j}^{o}$ for $0 \le i, j \le p - 1$ and $o \in \{T, L, D, R\}$ be as defined above. If $S_{c_{1}, \theta _{1}}$ and $S_{c_{2}, \theta _{2}}$ are two unit squares such that $c_{1}, c_{2} \in \triangle ABC$ then $S_{c_{1}, \theta _{1}} \cap S_{c_{2}, \theta _{2}} \ne \emptyset$.

### Proof

Lemma 2-1 shows that $l < 1$ and Lemma 2-5 gives that the base length of $\triangle _{i, j}^{o}$ is *l* while its legs have length $\frac{l\sqrt{2}}{2}$. The result follows from Lemma 3. $\square$

Let $K := 4 p^{2}$ be the number of triangles in the tiling. Proposition 3 states that we can split the SCSP into a set of $$\left( {\begin{array}{c}K\\ n\end{array}}\right) $$ subproblems. In each subproblem, we enforce that the center of each square belongs to a given triangle. For example, one can define the subproblem $T_{0}T_{19}T_{33}$ in the same tiling displayed in Fig. 3-Left. In this case, we set the standard constraint satisfaction problem defined in (1) and add to the model the linear inequalities given by Eqs. (8)–(11) for $\triangle _{0,0}^{T}$, $\triangle _{1, 1}^{R}$ and $\triangle _{2, 2}^{L}$ respectively.

We conclude this subsection by proving that several subproblems can be discarded without any processing due to symmetries in  and . Let $f^{90}, f^{180}, f^{270}: \mathbb {R}^{2} \rightarrow \mathbb {R}^{2}$ be the linear mappings that rotate the vector $x \in \mathbb {R}^{2}$ by an angle of 90, 180 and 270 degrees respectively. In the same way, define the linear mappings $f^{x}, f^{y}, f^{Id}, f^{-Id}: \mathbb {R}^{2} \rightarrow \mathbb {R}^{2}$ as the reflections around the lines $x = 0$, $y = 0$, $y = x$ and $y = -x$ respectively.

### Proposition 4

Let $\triangle _{i, j}^{o}$ for $0 \le i, j \le p - 1$ and $o \in \{T, L, D, R\}$ be a triangle of form (8) to (11). Then, $f^{op}(\triangle _{i,j}^{o})$ for $op \in \{90, 180, 270, r, x, Id, -Id\}$ is a triangle of form $\triangle _{i', j'}^{o'}$ with $0 \le i', j' \le p - 1$ and $o' \in \{T, L, D, R\}$.

### Proof

The triangle $\triangle _{i,j}^{o}$ has two vertices in  and one vertex in . Let *A* and *B* be the vertices in  and *C* be the vertex in . Lemma 2-3 ensures that  while Lemma 2-4 gives that . Since rotations and reflections are rigid transformations, the result holds. $\square$

Proposition 4 allows us to discard subproblems that are symmetric by rotations or reflections. For example, let $\overline{r}_{3} = \frac{5\sqrt{17}}{16}$ and $r_{3}, S_{c_{1}, \theta _{1}}, S_{c_{2}, \theta _{2}}, S_{c_{3}, \theta _{3}}$ be a feasible arrangement for (6) with $c_{1} \in T_{7}$, $c_{2} \in T_{12}$ and $c_{3} \in T_{22}$. Then, Proposition 4 ensures that there exists a feasible arrangement $r_{3}, S_{c_{1}', \theta _{1}'}, S_{c_{2}', \theta _{2}'}, S_{c_{3}', \theta _{3}'}$ satisfying $c_{1}' \in T_{19}$, $c_{2}' \in T_{12}$ and $c_{3}' \in T_{22}$. Moreover, since $T_{19}T_{12}T_{22}$ is obtained by a reflection around the *y* axis of $T_{7}T_{12}T_{22}$, we know that $c_{i}' = f^{y}(c_{i})$ for $i = 1, 2, 3$.

The tiling produced by Algorithm 2 suffices if one wants to use a complete global optimization approach for the packing problem. On the other hand, it is not suitable for a rigorous approach since the elements in  and  are floating point vectors subject to rounding errors. To overcome this problem, we introduce a scaled tiling. In this case, we ensure that the points at  and  are integer vectors to the cost of working with squares that are not unit but have the side length contained in a small interval $\mathbf{s}$. Algorithm 3 produces the scaled vertices for the tiling as well as the interval $\mathbf{s}$.
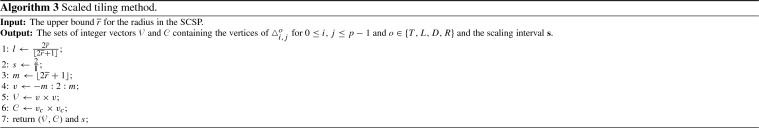


The elements in  and  are integer vectors by construction. Then, the Eqs. (8)–(11) are exactly representable. On the other hand, we replace the constant *s* in the Problem (6) by the interval $\mathbf{s}$ to keep the mathematical certainty of our statements. The lemmas and propositions in the last section remain valid after the proper scaling. Figure 3-Right illustrates the scaled tiling for $\overline{r}_{3} = \frac{5\sqrt{(17)}}{16}$. Note that the tiling would be the same for $\overline{r}_{4} = \sqrt{2}$ and the only difference between both cases would be the scaling interval $\mathbf{s}$.

Markót and Csendes [24] propose tiling constraints for the circle packing problem based on rectangles. The same idea could be used for the packing of squares into a circle. On the other hand for the case $n = 3$, one would need to split the search domain in 144 squares instead of 36 as proposed in this paper.

## Packing 3 unit squares

Friedman [9] gives an upper bound for the case $n = 3$, $\overline{r}_{3} = \frac{5 \sqrt{17}}{16}$. Algorithm 3 gives the tiling displayed in Fig. 3-Right and the interval scaling factor

12 $$\begin{aligned} s := [\underline{2.328342000348}79, \underline{2.328342000348}80]. \end{aligned}$$

**Fig. 4:**
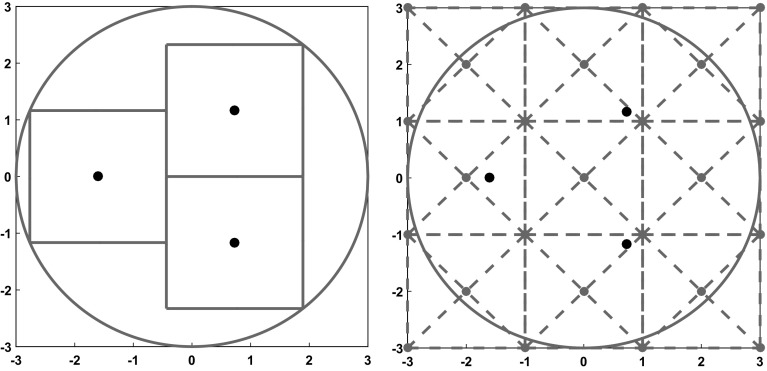
Left: An optimal configuration for $n = 3$. Right: Triangles 7, 12 and 22 contain an optimal arrangement

Figure 4-Left displays an optimal configuration associated to the scaled version of the problem. This section proves the theorem below.

### Theorem 2

Let $r_{3}$ be the solution of (1) for $n = 3$. Then,

$$\begin{aligned} r_{3} \in [\underline{1.288470508005}47, \underline{1.288470508005}53]. \end{aligned}$$

Moreover, the parameters of $S_{c_{1}, \theta _{1}}$, $S_{c_{2}, \theta _{2}}$ and $S_{c_{3}, \theta _{3}}$ belong to the boxes in Table 6.

### Proof

We perform the computational part of the proof in a *core i7* processor with a frequency of 2.6 GHz, 6 GB of RAM and *Windows 10*. We compiled the code using the *g++ 7.3* compiler with the option $-O3$. A supplementary material for the proof, containing the statistics and the log files for each subproblem is available in http://www.mat.univie.ac.at/~montanhe/publications/n3.zip

We prove the theorem in three phases. At the *i*-th iteration, we consider instances of form (6) and define subproblems by adding tiling constraints of form (8)–(11) accordingly.

The proof considers the scaled version of the problem to ensure the mathematical certainty of our statements. Therefore, the CSPs in this section are of form (6) with the constant *s* replaced by the interval $\mathbf{s}$ in Eq. (12). We obtain the unscaled interval for $r_{3}$ and Table 6 by dividing every box found in the last iteration by $\mathbf{s}$.

We also assume the labeling scheme for the triangles introduced in Sect. 4 and displayed on Fig. 3-Right. Therefore, the subproblem $T_{7}T_{12}T_{22}$ refers to the SCSP with the interval scaling factor $\mathbf{s}$ and such that $c_{1} \in T_{7} := \triangle _{0, 1}^{R}$, $c_{2} \in T_{12} := \triangle _{1, 0}^{T}$ and $c_{3} \in T_{22} := \triangle _{1, 2}^{D}$.

**Phase 1** In this iteration, we are interested in reducing the search domain of each subproblem and finding triangles which can contain the center of squares with no rotation. The tiling has 36 triangles, but the symmetries in  and  reduce the number of subproblems to 6. Table 2 shows the instances discarded without any processing in the first phase.

**Table 2:** Instances discarded in the first phase without processing

Instance	Symm. to	Symm. type	Instance	Symm. to	Symm. type
$T_{2}$	$T_{1}$	Ref. $y = x$	$T_{21}$	$T_{6}$	Rot. $90^{\circ }$
$T_{3}$	$T_{0}$	Ref. $y = x$	$T_{22}$	$T_{12}$	Rot. $180^{\circ }$
$T_{6}$	$T_{4}$	Ref. $x = 0$	$T_{23}$	$T_{6}$	Ref. $y = -x$
$T_{8}$	$T_{2}$	Ref. $x = 0$	$T_{24}$	$T_{3}$	Rot. $270^{\circ }$
$T_{9}$	$T_{2}$	Rot. $90^{\circ }$	$T_{25}$	$T_{3}$	Ref. $y = 0$
$T_{10}$	$T_{3}$	Rot. $90^{\circ }$	$T_{26}$	$T_{2}$	Ref. $y = 0$
$T_{11}$	$T_{3}$	Ref. $x = 0$	$T_{27}$	$T_{2}$	Rot. $270^{\circ }$
$T_{12}$	$T_{7}$	Rot. $270^{\circ }$	$T_{28}$	$T_{6}$	Rot. $180^{\circ }$
$T_{13}$	$T_{6}$	Ref. $y = x$	$T_{29}$	$T_{12}$	Rot. $270^{\circ }$
$T_{14}$	$T_{5}$	Rot. $270^{\circ }$	$T_{30}$	$T_{6}$	Ref. $y = 0$
$T_{15}$	$T_{6}$	Rot. $270^{\circ }$	$T_{31}$	$T_{14}$	Rot. $270^{\circ }$
$T_{17}$	$T_{16}$	Rot. $270^{\circ }$	$T_{32}$	$T_{2}$	Rot. $180^{\circ }$
$T_{18}$	$T_{17}$	Rot. $270^{\circ }$	$T_{33}$	$T_{3}$	Rot. $180^{\circ }$
$T_{19}$	$T_{17}$	Rot. $180^{\circ }$	$T_{34}$	$T_{3}$	Ref. $y = -x$
$T_{20}$	$T_{14}$	Rot. $180^{\circ }$	$T_{35}$	$T_{2}$	Ref. $y = -x$

Symm. to stands for symmetric to. Symm. type shows the operation needed to obtain Instance from the element in the second column

Instances $T_{0}$, $T_{1}$, $T_{4}$, $T_{5}$, $T_{7}$ and $T_{16}$ require processing. We run the Algorithm 1 with $\epsilon _{T} = 10^{-1}$, $\epsilon _{C} = 10^{-11}$, $\epsilon _{x} = 10^{-13}$ and time limit of 300 s. In this phase, we remove the condition $\theta _{1} = 0$ in Problem (6). Table 3 summarizes the results of the processed instances on phase 1. It shows that $T_{1}$ and $T_{5}$ are infeasible and any combination containing one of these triangles or their symmetric counterparts could be removed in the next phases. Moreover, it shows that only triangles $T_{7}$ and $T_{16}$ can contain the center of a square with rotation angle 0. Since we are assuming that $\theta _{1} = 0$ in the optimal configuration for $n = 3$, we only have to check the combinations containing at least one of these triangles.

**Table 3:** Statistics for the processed instances on phase 1

Instance	Status	Time(s)	Steps	${\theta }$
$T_{0}$	Timeout	300	428253	[0.38528, 1.21015]
$T_{1}$	Infeasible	1	1	-
$T_{4}$	Timeout	300	421457	[0.33751, 1.51529]
$T_{5}$	Infeasible	1	1	-
$T_{7}$	Timeout	300	398689	[0, 1.5708]
$T_{16}$	Timeout	300	336345	[0, 1.5708]

Status gives the termination status of the instance. Time(s) gives the processing time in seconds. Column steps displays the number of calls of the state machine described in Table 1. Column ${\theta }$ is a rigorous enclosure for the rotation angle

**Phase 2** This phase aims to discard as many instances as possible to reduce the number of hard subproblems in the last iteration. There are 630 possible combinations of 36 triangles taken 2 by 2. After eliminating symmetric and previously discarded cases, we obtain 43 instances. We also propagate any reduction in the search domain in the first phase to the subproblems in the second phase. Again, we remove the condition $\theta _{1} = 0$ from Problem (6).

We run the Algorithm 1 with $\epsilon _{T} = 10^{-1}$, $\epsilon _{C} = 10^{-11}$, $\epsilon _{x} = 10^{-13}$ and time limit of 3600 s. We stop the algorithm as soon as the feasibility verification method described in Sect. 2 succeeds in finding a feasible point. The supplementary material contains the list of all instances discarded without processing. Table 4 gives the statistics for the 43 processed instances.

**Table 4:** Statistics for the processed instances on phase 2

Instance	Status	Time(s)	Steps	Instance	Status	Time(s)	Steps
$T_{7}T_{17}$	Infeasible	1	17697	$T_{0}T_{28}$	Feasible	1	1
$T_{7}T_{19}$	Feasible	1	1	$T_{0}T_{29}$	Feasible	1	1
$T_{7}T_{29}$	Feasible	1	1	$T_{0}T_{30}$	Infeasible	1	11395
$T_{16}T_{17}$	Feasible	1	3	$T_{0}T_{33}$	Feasible	1	1
$T_{16}T_{18}$	Feasible	1	2	$T_{0}T_{34}$	Feasible	1	1
$T_{0}T_{3}$	Infeasible	1	81	$T_{4}T_{6}$	Infeasible	2	1603
$T_{0}T_{4}$	Infeasible	2	1211	$T_{4}T_{7}$	Infeasible	1	2657
$T_{0}T_{6}$	Infeasible	2	233	$T_{4}T_{12}$	Feasible	1	1
$T_{0}T_{7}$	Infeasible	2	2219	$T_{4}T_{13}$	Infeasible	1	13771
$T_{0}T_{10}$	Infeasible	1	861	$T_{4}T_{15}$	Feasible	1	1
$T_{0}T_{11}$	Infeasible	2	1059	$T_{4}T_{16}$	Infeasible	1	24983
$T_{0}T_{12}$	Infeasible	1	2527	$T_{4}T_{17}$	Infeasible	1	4923
$T_{0}T_{13}$	Infeasible	1	243	$T_{4}T_{18}$	Feasible	1	1
$T_{0}T_{15}$	Infeasible	2	1535	$T_{4}T_{19}$	Feasible	1	1
$T_{0}T_{16}$	Feasible	1	1	$T_{4}T_{21}$	Infeasible	1	705
$T_{0}T_{17}$	Infeasible	1	3397	$T_{4}T_{22}$	Infeasible	2	19323
$T_{0}T_{18}$	Infeasible	1	3889	$T_{4}T_{28}$	Feasible	1	1
$T_{0}T_{19}$	Feasible	1	1	$T_{4}T_{29}$	Feasible	1	1
$T_{0}T_{21}$	Infeasible	1	8021	$T_{4}T_{30}$	Feasible	1	1
$T_{0}T_{22}$	Feasible	1	1	$T_{7}T_{12}$	Feasible	1	1
$T_{0}T_{23}$	Feasible	1	1	$T_{7}T_{16}$	Feasible	1	1
$T_{0}T_{24}$	Infeasible	2	1227				

Status gives the termination status of the instance. Time(s) gives the processing time in seconds. Column steps displays the number of calls of the state machine described in Table 1

We conclude the second phase with 22 infeasible subproblems. Again, any case in the next phase containing a combination found infeasible in this step can be discarded without any processing.

**Phase 3** In this phase we set the full model in Problem (6), including the constraint $\theta _{1} = 0$. Table 3 shows that $c_{1} \in T_{7}$ or $c_{1} \in T_{16}$. Therefore, after removing the cases where one of these conditions do not hold and eliminating symmetric and already proved infeasible subproblems, we obtain 12 instances of the 7140 possible ones.

If an instance contains both triangles $T_{7}$ and $T_{16}$, we denote by $T_{7*}T_{16}T_{x}$ the case where we enforce the angle of the square centered in $T_{7}$ to be zero. In the same way, we write $T_{7}T_{16*}T_{x}$ for the instances where the square centered in $T_{16}$ has no rotation angle.

For the last phase, we run Algorithm 1 with $\epsilon _{T} = 10^{-1}$, $\epsilon _{C} = 10^{-11}$, $\epsilon _{x} = 10^{-13}$ and no time limit. Table 5 provides the statistics of the processed instances. Moreover, Table 5 shows that it is the only instance containing the optimal configurations for $n = 3$. Figure 4-Right shows an approximation of the center of each square in the optimal case.

Algorithm 1 produces 4 clusters for the instance $T_{7}T_{12}T_{22}$. The maximum width of a cluster is $6.23*10^{-13}$. The precision is smaller than $\epsilon _{x}$ due to the cluster builder procedure described in Sect. 2. Table 6 gives the unscaled clusters.$\square$

**Table 5:** Statistics for the processed instances on phase 3

Instance	Status	Time(s)	Steps
$T_{7*}T_{12}T_{16}$	Infeasible	33	146629
$T_{7}T_{12}T_{16*}$	Infeasible	134	440307
$T_{7}T_{12}T_{22}$	Clusters found	628	2183739
$T_{7*}T_{16}T_{18}$	Infeasible	3	16901
$T_{7}T_{16*}T_{18}$	Infeasible	3	18273
$T_{7*}T_{16}T_{19}$	Infeasible	4	19729
$T_{7}T_{16*}T_{19}$	Infeasible	1	5491
$T_{7*}T_{16}T_{29}$	Infeasible	8	40345
$T_{7}T_{16*}T_{29}$	Infeasible	2	11071
$T_{16}T_{17}T_{18}$	Infeasible	2	7319
$T_{0}T_{16}T_{19}$	Infeasible	0	2833
$T_{0}T_{16}T_{29}$	Infeasible	2	9317

Status gives the termination status of the instance. Time(s) gives the processing time in seconds. Column steps displays the number of calls of the state machine described in Table 1

**Table 6:** Enclosures of the optimal arrangement for $n = 3$

**Square**	$\mathbf{c}_{1}$	$\mathbf{c}_{2}$	${\theta }$
1	$[-0.68750000000001, -0.68749999999988]$	$[-0.00000000000018, 0.00000000000023]$	[0.0, 0.0]
2	[0.31249999999993, 0.31250000000014]	$[-0.50000000000007, -0.49999999999989]$	[1.57079632679426, 1.57079632679491]
3	[0.31249999999993, 0.31250000000011]	[0.49999999999992, 0.50000000000007]	$[-0.00000000000001, 0.00000000000050]$
1	$[-0.68750000000001, -0.68749999999988]$	$[-0.00000000000021, 0.00000000000012]$	[0.0, 0.0]
2	[0.31249999999998, 0.31250000000019]	$[-0.50000000000003, -0.49999999999990]$	$[-0.00000000000001, 0.00000000000036]$
3	[0.31249999999993, 0.31250000000009]	[0.49999999999995, 0.50000000000007]	$[-0.00000000000001, 0.00000000000029]$
1	$[-0.68750000000001, -0.68749999999991]$	$[-0.00000000000012, 0.00000000000016]$	[0.0, 0.0]
2	[0.31249999999993, 0.31250000000011]	$[-0.50000000000007, -0.49999999999993]$	[1.57079632679463, 1.57079632679491]
3	[0.31249999999998, 0.31250000000014]	[0.49999999999993, 0.50000000000004]	[1.57079632679453, 1.57079632679491]
1	$[-0.68750000000001, -0.68749999999992]$	$[-0.00000000000014, 0.00000000000014]$	[0.0, 0.0]
2	[0.31249999999998, 0.31250000000011]	$[-0.50000000000003, -0.49999999999995]$	$[-0.00000000000001, 0.00000000000027]$
3	[0.31249999999998, 0.31250000000011]	[0.49999999999995, 0.50000000000004]	[1.57079632679463, 1.57079632679491]

There are 4 clusters, each of them separated by a blank line. The first coordinate of the center of the *i*-th square is given by $\mathbf {c_{1}}$ and the second coordinate by $\mathbf {c_{2}}$. The rotation angle is given by ${\theta }$

## Conclusion

This paper presents a framework for the rigorous optimization of the packing of unit squares into a circle. We express the question as the standard constraint satisfaction problem stated by Definition 1. The model considers the concept of sentinels to formulate non-overlapping constraints and the convexity of the squares and the circle to describe containment conditions.

General purpose rigorous optimization solvers cannot achieve the solution of the standard constraint satisfaction problem due to symmetries in the search domain. To overcome this difficulty, we propose a tiling method that splits the search space related to the center of each unit square into isosceles triangles. Our tiling divides the original problem into a set of subproblems that are suitable for the interval branch-and-bound approach. We also ensure that the parameters in each subproblem are free of rounding errors by introducing a proper scaling of the search domain.

To show the capabilities of our approach, we solve the first open case reported in the literature, $n = 3$. We implement the interval branch-and-bound in the *C++* and the code is publicly available. We perform the proof on an ordinary laptop with 6 GB of RAM and a core *i7* processor.

The proof of the case $n = 3$ requires the solution of 6 subproblems with one square, 43 with two and only 12 with three squares. We discard most subproblems without processing due to symmetries in the tiling. Among the 61 subproblems, just 6 require more than 100 s to conclude the search. At the end of the process, we obtained 4 boxes with the following properties

1. The maximum width of any coordinate of the resulting boxes is $6.23*10^{-13}$.
2. If one disregard symmetries, every solution of (1) is contained in at least one of the 4 boxes

The method proposed in this paper could, in principle, be used to find the optimal arrangement for higher values of *n* (e.g., $n = 4, 5, 6$.).

## Electronic supplementary material

Below is the link to the electronic supplementary material.

Supplementary material 1 (zip 65514 KB)
